# Radiation doses and estimated risk from angiographic projections during coronary angiography performed using novel flat detector

**DOI:** 10.1120/jacmp.v17i3.5926

**Published:** 2016-05-08

**Authors:** Anna Varghese, Roshan S. Livingstone, Lijo Varghese, Parveen Kumar, Sirish Chandra Srinath, Oommen K. George, Paul V. George

**Affiliations:** ^1^ Department of Radiology Christian Medical College Vellore TamilNadu, South India; ^2^ Department of Cardiology Christian Medical College Vellore TamilNadu, South India

**Keywords:** coronary angiography, angiographic projections, radiation dose, effective dose

## Abstract

Coronary angiography (CA) procedure uses various angiographic projections to elicit detailed information of the coronary arteries with some steep projections involving high radiation dose to patients. This study intends to evaluate radiation doses and estimated risk from angiographic projections during CA procedure performed using novel flat detector (FD) system with improved image processing and noise reduction techniques. Real‐time monitoring of radiation doses using kerma‐area product (KAP) meter was performed for 140 patients using Philips Clarity FD system. The CA procedure involved seven standard projections, of which five were extensively selected by interventionalists. Mean fluoroscopic time (FT), KAP, and reference air kerma (Ka,r) for CA procedure were 3.24 min (0.5–10.51), 13.99 Gycm^2^ (4.02–37.6), and 231.43 mGy (73.8–622.15), respectively. Effective dose calculated using Monte Carlo‐based PCXMC software was found to be 4.9 mSv. Left anterior oblique (LAO) 45° projection contributed the highest radiation dose (28%) of the overall KAP. Radiation‐induced risk was found to be higher in females compared to males with increased risk of lung cancer. An increase of 10%–15% in radiation dose was observed when one or more additional projections were adopted along with the seven standard projections. A 14% reduction of radiation dose was achieved from novel FD system when low‐dose protocol during fluoroscopy and medium‐dose protocol during cine acquisitions were adopted, compared to medium‐dose protocol.

PACS number(s): 87.50.cm, 87.55.de, 87.55.N, 87.59.cf, 87.59.Dj

## I. INTRODUCTION

Cardiovascular disease (CVD) accounts for nearly half of the noncommunicable diseases leading to the global cause of mortality.^(1)^ With increased pace of technological advancements, minimally invasive image‐guided cardiovascular interventions are gaining importance across various health‐care centers for the diagnosis of CVDs. Cardiovascular interventions, which predominantly include coronary angiography (CA) and percutaneous transluminal coronary angioplasty (PTCA), are performed using dedicated fluoroscopic systems equipped with either flat detector (FD) or image intensifier (II). These machines have the potential to either impart high or low radiation doses to patients depending on the protocols selected by the interventionalist.

Radiation doses from PTCA may reach threshold doses of the order of 2 Gy with the ability of inducing deterministic effect and increased stochastic risk.^2^, ^3^, ^4^ Though deterministic effects are not commonly reported from CA procedures, it can elevate the radiation induced risks, with its effective dose ranging from 3–10 mSv.^2^, ^5^ The new generation FD systems with advanced image processing technology allows acquisition of images with reduced dose whilst maintaining adequate image quality.^(6)^ An alternative to the conventional CA is the cardiac‐computed tomography angiography (CTA) examination which has an effective dose ranging from 5–32 mSv.^(7)^ Recent studies have shown that dose from CTA using modern CT scanners may deliver an effective dose of 2.5 mSv.^(8)^ Though noninvasive CTA is performed in various health‐care centers, conventional CA still continues to be the standard for diagnosing CVD.^(9)^

Radiation doses imparted to patients during angiography are influenced by several factors such as body mass index (BMI) of the patient, fluoroscopic time, dose rates, tube angulations/projection, and number of cine acquisitions.^10^, ^11^ It is reported that CA procedures involving oblique projections coupled with shallow or steep cranial or caudal angulation would help in visualizing cardiac vessels distinctly without any overlap, thus providing sufficient reference image for intervening the lesions.^12^, ^13^, ^14^ These steep projections involve increased radiation dose to both staff and patients. There is an increased number of interventions performed annually,^(15)^ and contribution of radiation doses from individual projections is not widely reported in clinical setup to the extent of providing awareness among interventionalists as the study protocols may vary. However, there are studies in literature on radiation doses from various projections on anthropomorphic phantoms.^(16)^ Hence it is necessary to monitor radiation doses and their associated risks during cardiovascular interventions. The kerma‐area product (KAP) displayed in the console of an interventional suite is a dose descriptor which serves as an useful tool to assess radiation dose from interventional procedures and acts as a surrogate for radiation risk.^(17)^ There is very little information on radiation dose from novel FD systems in literature. This study aims to monitor KAP and estimate radiation risk from each angiographic projection during CA performed using novel FD system. It is anticipated that this information would be valuable for creating awareness on radiation doses from CA procedures among practicing interventional cardiologists.

## II. MATERIALS AND METHODS

The study was approved by the institutional review board of the institution (IRB minute No. 8805, 2013). Radiation doses and exposure factors from various projections were acquired for 140 coronary angiograms which spanned for a study period of five months. All interventions were performed in a catheterization lab equipped with Philips Allura Clarity FD10 (Eindhoven, The Netherlands) system with enhanced image processing chain and noise reduction techniques for effective dose reduction. The 25 cm detector had pixel dimensions of 184×184 μm with detective quantum efficiency (DQE): >75% at low spatial frequencies. The system had tube potentials ranging from 40 to 125 kVp with maximum tube current of 1062 mA. For measurement of reference air kerma, the distance between focal spot to the patient entrance surface reference point was 61.5 cm. The measured air‐kerma rates for 25 cm detector format was 0.159, 0.24, and 0.55 mGy/s for low, medium, and normal fluoroscopy modes, respectively. An inherent filtration of 2.5 mm Al, and spectral filters of 0.4 mm Cu for low and medium dose rate and 0.1 mm Cu for normal dose rate were integrated in the system during fluoroscopy. During cine acquisitions, 0.4 mm Cu and 0.1 mm Cu filters were selected for low and medium dose rates, respectively.

### A. Dosimetry

Radiation doses were measured using calibrated KAP meter (DIAMENTOR, PTW; Freiburg, Germany) fitted on the top of the collimator assembly. Periodic calibration of the KAP meter was made using Unfors Xi multiparametric QA kit (Unfors Instruments, AB, Billdal, Sweden). During CA, real‐time monitoring of radiation doses and exposure factors such as tube potential (kV), tube current (mA), fluoroscopy time, and number of cine acquisition for each angiographic projection were elicited by personnel involved in data collection. Reference air‐kerma (Ka,r) values which serves as a dose indicator for deterministic effects were estimated at the interventional reference point (IRP).^(18)^ In order to estimate gender‐based radiation risks, effective dose (ED) and organ doses (breasts, heart, lungs) and radiation exposure‐induced cancer death (REID) associated to cardiovascular imaging were calculated using PC‐based Monte Carlo simulation software (PCXMC) developed by STUK (Radiation and Nuclear Safety Authority, Helsinki, Finland).^(19)^ The PCXMC software calculates organ doses and effective doses (ED) based on tissue weighting factors according to International Commission on Radiation Protection (ICRP) 103, and estimates potential cancer risks based on models used in BEIR VII (Biological Effects of Ionizing Radiations).^20^, ^21^ The calculation involved input of patient size, beam projection, source to image distance (SID), tube potentials, anode angle, and total beam filtration. In this study, estimates of uncertainty associated with REID were from the Asian mortality data available in the PCXMC software.

### B. Coronary angiography procedure

CA is an invasive diagnostic procedure performed to visualize contrast filled‐blood vessels of the coronaries under fluoroscopic guidance. The procedure was performed by senior cardiology residents assisted by junior consultants. A radial artery approach using dedicated TIG diagnostic five French catheters Terumo International Systems, Somerset, NJ) for left and right coronary angiography were selected in the study. A 25 cm FD format was used to trace the path of the catheter from the arm to the coronaries and a magnification of 20 cm for the rest of the procedure. Cine acquisitions were acquired at a rate of 15 frames per second (fps) and the SID varied from 95 cm to 120 cm, depending on tube angulations. Imaging of coronaries were performed with left anterior oblique (LAO) and right anterior oblique (RAO) projections coupled with either cranial (CRA) or caudal (CAU) angulations. Table 1 shows seven standard and additional angiographic projections to visualize left and right coronary arteries followed by all interventionalist.^(22)^ For patients with dominant right coronary artery (RCA), three projections (LAO 45°, LAO10°‐CRA25°, and RAO 30°) were standard, of which, RAO 30° was avoided when dominant RCA was normal. In case of codominant RCA, cine acquisition was restricted to LAO 45°. Additional projections were selected specifically to view the lesion of the left coronaries. Among these projections, the most common were PA‐CAU 40° and RAO 40°‐ CRA 40°; while RAO 35°‐CAU 35° and LAO 90° were sparingly selected. Evaluation of image quality analysis using ImageJ software (National Institute of Health, Bethesda, MD) was performed for low and medium dose rate fluoroscopic images and their signal to noise ratio (SNR) were calculated using the formula:^(23)^

(1) $$SNR=\frac{\Delta I}{\sigma_{I}}$$

where ΔI is the change in intensity caused by lesion or region of interest, and $\sigma_{I}$ is the standard deviation of background intensity due to noise.

**Table 1:** Angiographic projections for coronary angiography procedure.

*Coronary System*	*Standard Projections*	*Region of Interest*	*Additional Projections*	*Region of Interest*
Left coronary artery	RAO 20°‐CAU 20°	Left main, proximal LAD, proximal LCX	PA‐CAU 40°	Left main, proximal LAD, proximal LCX
	RAO 10°‐CRA 40°	Mid and distal LAD, diagonals	RAO 35°‐CAU 35°	Left main, proximal LAD, proximal LCX
	LAO 45°‐CRA 15°	Mid and distal LAD in orthogonal plane	RAO 40°‐CRA 40°	Mid and distal LAD
	LAO 45°‐CAU 35°	Left main, proximal LCX	LAO 90°	Mid and distal LAD
Right coronary artery	LAO 45°	Proximal and mid RCA	LAO 90°	Proximal and mid RCA
	LAO 10°‐CRA 25°	Distal RCA, bifurcation into PDA and posterolateral branches		
	RAO 30°	Mid RCA		

LAD=left anterior descending artery; LCX=left circumflex; RCA=right coronary artery; PDA=posterior descending artery; LAO=left anterior oblique; RAO=right anterior oblique; CAU=caudal; CRA=cranial.

## III. RESULTS

Out of the 140 patients, 98 were males and 42 were females with an average body mass index (BMI) of 24.29 and $25.85\;{\text{kgm}}^{-2}$, respectively. During CA, tube potentials ranged from 75 to 108 kV with tube current varying between 7.4 and 20.8 mA depending on the dose rate selected. The mean fluoroscopic time duration was 3.24 min (0.5‐10.51), with KAP and Ka,r of 13.99 Gycm^2^ (4.02‐37.6) and 231.43 mGy (73.8‐622.15), respectively. The mean number of cine acquisitions was 7.3 (4‐14) with an average of 525 frames (246‐1063). The LAO 45° projection involved maximum KAP of 4.85 Gycm^2^ due to prolonged fluoroscopic screening and cine run to view the right coronary artery (RCA) when compared to other projections. However, the contribution of KAP values from cine acquisitions was found to be maximum in LAO 45° ‐ CAU 35° projection (Table 2). The ED estimated from angiographic projections showed that LAO 45° contributed to the maximum ED of 1.74 mSv, followed by LAO45°‐CAU35° and PA‐CAU40° with ED of 0.78 and 0.52 mSv, respectively. The sum of the effective dose from all angiographic projections for CA procedure was found to be 4.9 mSv (Table 2).

The ED from fluoroscopy and cine acquisitions for frequently used dose rate is shown in Table 3. ED was estimated for 29 organs and tissues using Monte Carlo‐based software. In our study, organs such as breast, heart, and lungs were reported as they received considerable amount of radiation doses compared to the other organs. The KAP values from cine acquisitions were higher than fluoroscopic screening, while the ED was low. This was due to lower tube potentials and filtration used during cine acquisition, and the estimation of ED using PCXMC software was based on these factors. The radiation exposure‐induced cancer death (REID) for female patients was higher than males with high risk of lung cancer in both sexes compared to other organs such as heart and breast. Selection of low dose rate in both fluoroscopy and cine acquisitions resulted in a dose reduction of approximately 52% compared to the medium dose rate (Table 4). The SNR for low and medium dose rate fluoroscopy images were 4.09 and 5.65, respectively.

**Table 2:** Exposure factors and dose information for various angiographic projections.

		*Fluoroscopic Screening* Mean±SD (range)				*Cine Acquisition 15 fps* Mean±SD (range)					*Total KAP (Gycm^2^) Mean* Mean±SD (range)	*Effective Dose (mSv)*
*Angiographic Projections*	*N*	*kV*	*mA*	*FT (min)*	*KAP (Gycm^2^)*	*kV*	*mA*	*Number of Cine Runs*	*Frames*	*KAP (Gycm^2^)*		
PA	126	75±4.6 (61‐90)	11±3.8 (4.1‐19)	0.36±0.5 (0.01‐2.53)	0.42±0.35 (0.009‐2.1)	55	87	1 (0‐1)	0‐33	0.018	0.44±0.35 (0.009‐2.1)	0.13
RAO 20°‐CAU 20°^a^	140	80±6 (71‐102)	13.7±4.2 (7‐21)	0.18±0.3 (0.01‐2)	0.24±0.3 (0.01‐2.5)	73±2.8 (67‐80)	703±123 (448‐922)	1.13 (1‐3)	83±27 (43‐180)	0.8±0.5 (0.14‐3)	1.04±0.62 (0.19‐3.4)	0.31
RAO 10°‐CRA 40°^a^	138	84±8.2 (72‐108)	14.9±4.5 (6.9‐21)	0.1±0.2 (0.01‐0.82)	0.12±0.13 (0.01‐0.95)	77±5.1 (67‐100)	827±95 (423‐928)	1.09 (1‐3)	84±27.78 (34‐204)	1.17±0.75 (0.24‐5.5)	1.29±0.8 (0.27‐5.63)	0.26
LAO 45°‐CRA 15°^a^	86	90±10.4 (74‐118)	14.5±4.8 (6.3‐21.1)	0.09±0.2 (0.01‐1.06)	0.18±0.17 (0.03‐1.11)	81±5.8 (72‐96)	848±57.8 (670‐924)	1.06 (1‐3)	75±21.7 (16‐152)	1.45±0.7 (0.2‐3.8)	1.63±0.75 (0.41‐4.08)	0.40
LAO 45°‐CAU 35°^a^	140	110±10.3 (85‐120)	14.2±5 (6.9‐21.3)	0.15±0.2 (0.01‐1.38)	0.47±0.57 (0.03‐3.56)	95±10.9 (73‐125)	758±81.7 (572‐926)	1.07 (1‐4)	71±23 (30‐179)	2.2±1.18 (0.32‐7.5)	2.67±1.47 (0.4‐10.64)	0.78
LAO 45°^a^	140	88±9.6 (74‐115)	13.45±4.7 (6.2‐21.1)	2.19±1.9 (0.22‐10.01)	3.77±3.16 (0.34‐14.65)	76±3.9 (69‐91)	799±103 (517‐928)	1.14 (1‐4)	80±40 (34‐275)	1.08±0.8 (0.13‐6.14)	4.85±3.5 (0.6‐16.54)	1.74
LAO 10°‐CRA 25°^a^	113	84±6.8 (73‐109)	13.8±4.4 (6.9‐20.9)	0.07±0.1 (0.01‐0.68)	0.11±0.15 (0.01‐1.05)	75±2.96 (67‐84)	791±98.2 (462‐928)	1.07 (1‐2)	76±27.5 (31‐190)	0.96±0.5 (0.23‐2.73)	1.07±0.52 (0.3‐2.88)	0.27
RAO 30°^a^	71	79±4.6 (73‐94)	13.5±4.2 (6.7‐20.9)	0.09±0.3 (0.01‐2.22)	0.11±0.21 (0.02‐1.7)	71±2.06 (67‐75)	632±96.9 (431‐847)	1.03 (1‐2)	77±38.5 (23‐196)	0.66±0.46 (0.09‐2.77)	0.77±0.54 (0.11‐2.91)	0.21
PA‐CAU 40°	80	101±13 (75‐120)	14.2±5 (7.6‐21.4)	0.13±0.2 (0.01‐1.21)	0.32±0.33 (0.03‐1.7)	84±8.9 (70‐106)	821±79.2 (567‐928)	1.04 (1‐3)	71±24 (27‐149)	1.5±0.9 (0.21‐4.64)	1.82±1.02 (0.42‐5.03)	0.52
RAO 40°‐CRA 40°	25	91±12.3 (70‐110)	14.2±5.1 (8.2‐21.1)	0.21±0.2 (0.02‐0.67)	0.28±0.26 (0.03‐0.93)	80±6.95 (69‐92)	819±101.6 (542‐928)	1.083 (1‐3)	76±27.7 (43‐154)	1.28±0.7 (0.23‐2.85)	1.56±0.78 (0.26‐3.11)	0.31

a Seven standard angiographic projections.

PA, Posterior‐Anterior; LAO, Left anterior oblique; RAO, Right anterior oblique; CAU, Caudal; CRA, Cranial; KAP, Kerma area product; FT, Fluoroscopic time; SD, Standard deviation.

**Table 3:** Gender‐based effective doses, organ doses, and radiation exposure‐induced cancer death (REID) for low and medium dose rates.

			*Fluoroscopic Screening*						*Cine Dose Rate*	*Cine Acquisition*		
					*Organ Dose (mGy)*							*Organ Dose (mGy)*				*Total*	
*Fluoroscopy Dose Rate*	*Gender*	*N*	*KAP (Gycm^2^)*	*ED (mSv)*	*Breast*	*Heart*	*Lungs*	*REID (%)*		*KAP (Gycm^2^)*	*ED (mSv)*	*Breast*	*Heart*	*Lungs*	*REID (%)*	*ED (mSv)*	*REID (%)*
Medium	Male	44	7.61	2.76	1.56	7.07	10.41	0.013	Medium	13.03	3.05	1.29	6.69	10.01	0.014	5.82	0.026
	Female	21	6.12	2.46	1.48	6.6	9.79	0.02		8.03	2.2	1.03	5.19	8.5	0.018	4.66	0.038
Low	Male	37	4.61	1.54	1.27	3.71	5.97	0.007	Medium	12.56	2.73	1.46	5.56	9.21	0.012	4.28	0.019
	Female	11	5.57	2.51	1.23	8.54	11.09	0.021		9.39	2.38	0.92	5.14	8.27	0.017	4.88	0.037

KAP=kerma‐area product; ED=effective dose; REID=radiation exposure induced death (Stochastic radiation risk estimate from ICRP 103 for Asian mortality data).

**Table 4:** Exposure factors and radiation dose for different dose rates.

*Protocol*			*Mean Exposure Factors* Mean±SD (range)			*Radiation Doses* Mean±SD (range)	
*Fluoroscopic Screening*	*Cine Acquisition*	*N*	*kV*	*mA*	*FT (min)*	*KAP (Gycm^2^)*	*Ka,r (mGy)*
Medium	Medium	74	86±6.5	17±1.8	3.30±2.2	15.41±6.7	255.01±107.5
			(75‐104)	(12‐20.8)	(0.5‐10.51)	(5.16‐37.59)	(82.79‐622.15)
Medium	Low	6	88±6.6	16.9±1.06	3.18±1.8	10.74±5.7	168.35±83.94
			(80‐99)	(14.64‐18.88)	(1.12‐5.59)	(5.79‐21.26)	(90.18‐326.95)
Low	Medium	52	90±6.5	9.2±1	3.27±2.2	13.33±5.4	220.73±81.84
			(76‐108)	(7.4‐12.6)	(1.15‐10.33)	(4.52‐32.91)	(75.77‐512.18)
Low	Low	8	89±6.2	9.5±0.7	2.53±1.6	7.31±2.2	126.41±32.22
			(80‐96)	(8.8‐10.6)	(1.17‐6.09)	(4.02‐9.78)	(73.76‐162.29)

FT=fluoroscopic time; KAP=kerma‐area product; Ka,r=reference air kerma; SD=standard deviation.

## IV. DISCUSSION & CONCLUSIONS

Diagnostic CA involves fluoroscopic screening and cine acquisitions, with cine acquisitions being the major contributor to the overall radiation dose. In general, during a CA procedure, seven standard angiographic projections are adopted to visualize left and right coronary arteries.^(22)^ In our study, out of the seven standard projections, five were extensively used. Furthermore, additional projections were selected depending on the pathology of the patient (Table 1). The percentage contribution of radiation dose from fluoroscopic screening and cine acquisitions in LAO, RAO, and PA projections were 70%, 27%, and 3%, respectively. LAO 45° being a standard projection contributed the highest radiation dose (28%) from the overall KAP compared to other projections. This was due to longer fluoroscopy duration involved in engaging the catheter into the left and right system in addition to cine run. Similar finding has been reported by Farajollahi et al.^(24)^ using LAO40° compared to LAO40°‐CAU10° and LAO40°‐CRA10°. Contrary to our findings, it is reported that the maximum contribution of overall KAP during CA procedure was from RAO projection compared to LAO, LAT, and PA projections.^25^, ^26^ It is known that the use of steep angulations involve longer SID, thus imparting higher radiation doses to patients. In our study, the contribution of radiation doses to the overall KAP from LAO45°‐CAU35° and PA‐CAU40° were 16% and 11%, respectively, due to high tube potential of 120 kV and tube current of 21.4 mA compared to other angiographic projections (Table 2). Kuon et al.^(16)^ reports that LAO45°‐CAU35° involves a 2.6 fold increase in KAP/sec and 7.6 fold increase in operator doses compared to PA‐CAU30°. However these measurements were extensively carried out using an anthropomorphic RANDO phantom.

In order to provide ED for a standard CA procedure, male and female patients who underwent nine angiographic projections (Table 2) were included. For an Asian population with average age of 50 yrs (40–70 yrs), the REID for male and female patients was 0.045% and 0.075%, respectively. Taking into consideration lung as the organ which received maximum radiation dose, the estimated radiation risk for lung cancer was 0.03% in males and 0.06% in females as assessed using PCXMC 2.0. Radiation‐induced breast cancer risk among females was 0.004% using medium dose rate and 0.0013% using low dose rate. The mean Ka,r was 231.43 mGy (73.8–622.15) for the CA procedure. From this study, the K (a,r) of 0.6 Gy was found to be within the threshold dose limit for inducing skin erythema which is 2 Gy.

During the study, interventionalists frequently used medium dose rate for both fluoroscopy and cine acquisitions. Radiation doses from CA can be optimized by selecting appropriate dose rate depending on the table top doses. Dose reduction of the order of 50% could be achieved with the use of low dose rate compared to medium dose rate (Table 4). During the study, interventionalists suggested the use of low dose rate for fluoroscopy and medium dose rate during cine acquisitions which involved a dose reduction of 14% compared to the use of medium dose rates during fluoroscopy. The percentage difference of SNR between low and medium dose rate fluoroscopic images was 32%. Tsapaki et al.^(27)^ reports a minimum KAP of 6.1 Gycm^2^ from FD system for CA with the use of low dose rate to optimize radiation doses. In our study, the lowest dose reported was 4.02 Gycm^2^ with the use of low dose setting for both fluoroscopy and cine acquisition. It should be noted that the KAP values also depend on patient size, skill and experience of the operator, imaging protocol, and equipment functionalities. In comparison to II and FD systems as reported by Livingstone et al.,^(28)^ the present study shows that there is a reduction of 50% and 43% of radiation doses during cine acquisitions with the use of novel FD showed. The dose reduction reported in our study was similar to the study performed by Dekker et al.,^(6)^ suggesting that advanced image processing and real‐time noise reduction algorithm has the potential to reduce radiation doses without compromising image quality.

With a concern of work practices, an increase in number of cine acquisitions and frames per projection will contribute to an increase of radiation dose. In our study, a maximum of three to four cine acquisitions were acquired for certain projections in a few patients due to complications during the procedure. Kuon et al.^(29)^ was able to establish a KAP of 6.2 Gycm^2^ from the II system with the use of low‐dose fluoroscopy by restricting the mean number of frames during cine acquisitions to 171, per CA procedure. Owing to concerns on radiation doses from interventional procedures, diagnostic reference levels (DRL) are suggested. In their study, Dragusin et al.^(30)^ report a reference level for FT, KAP, and Ka,r of 5 min, 23 Gycm^2^, and 376 mGy, respectively, from FD system. From our study, the third quartile KAP values and Ka,r was 16.76 Gycm^2^ and 285.91 mGy, respectively, for FT of 4.02 min for nine commonly used angiographic projections with a maximum of 12 cine acquisitions.

Adhering to seven standard angiographic projections (Table 1) would have a dose reduction of approximately 10%–15% compared to the additional projections obtained due to the complexity of the procedure. Among the frequently used projections, LAO 45° contributed to the maximum dose. The novel FD system with better image processing chain and dose reduction algorithm has the potential to reduce of radiation doses especially during cine acquisitions. To maintain optimal dose and image quality, it is suggested to use low dose rate during fluoroscopic screening and medium dose rate during cine acquisitions. Radiation induced risks was high for lungs compared to the other organs including the breast tissue in female population.

## COPYRIGHT

This work is licensed under a Creative Commons Attribution 4.0 International License.
